# Myelin Lipid Reserves as a Conditional Metabolic Buffer: Implications for Alzheimer’s Disease and Ischemic Stroke

**DOI:** 10.1007/s12017-026-08905-0

**Published:** 2026-01-30

**Authors:** Peibin Zou, Zhihai Huang, Yulan Zhang, Xuemei Zong, Quanguang Zhang

**Affiliations:** 1 Institute for Cerebrovascular and Neuroregeneration Research (ICNR), Department of Neurology, Louisiana State University Health Sciences Center, 1501 Kings Highway, Shreveport, LA 71103 USA; 2 Department of Pharmacology, Toxicology & Neuroscience, Louisiana State University Health Sciences Center, 1501 Kings Highway, Shreveport, LA 71103 USA

**Keywords:** Oligodendrocytes, Myelin, Lipid metabolism, Metabolic buffering, Beta oxidation, White matter energetics, Alzheimer disease, Ischemic stroke

## Abstract

Brain energetics rely on a distributed partnership among cell types and fuel sources. Beyond astrocytic glycogen, the brain has limited conventional energy reserves. Emerging evidence broadens this view by positioning myelin and oligodendrocytes as active stabilizers of metabolic homeostasis. They align substrate delivery with demand and directly sustain axonal ATP production. This review highlights current understanding that myelin lipid stores function as a conditional metabolic buffer that can be mobilized when glycolytic supply wanes. Firstly, we outline the protective repertoire of myelin (e.g., adaptive myelination, antioxidant defense, and metabolic coupling) and then summarize myelin lipid metabolism, spanning de novo synthesis and β-oxidation. We next demonstrate disease contexts marked by energetic failure. Specifically, Alzheimer’s disease exhibits a chronic metabolic downshift, whereas ischemic stroke produces an acute collapse of energy production. Both states may recruit the proposed buffer. However, leveraging lipid-derived fuels is not without risk. Reactive oxygen species, acidosis, and iron handling must be tightly regulated to avoid collateral injury. Finally, we highlight methodological priorities that can resolve mechanism in vivo, including white matter-resolved fluxomics, myelin specific imaging paired with proteo-lipidomics, and lineage-restricted perturbations of β-oxidation and autophagy. On the translational front, we propose stage specific strategies. In summary, defining when and how to mobilize and supplement myelin lipid reserves could transform a conceptual buffer into a practical lever for disease modification in hypometabolic brain disorders.

## Introduction

Neural information processing is energetically expensive (Attwell & Laughlin, 2001). In brain, tight coupling between substrate delivery, mitochondrial ATP production, and local removal of metabolic by-products is essential to sustain axonal signaling and prevent excitotoxic and oxidative injury (Sheng, 2017; X. T. Cheng et al., 2022). White matter has long been cast as a passive insulator that merely accelerates conduction. However, converging evidence now reframes myelin and its producing cells, oligodendrocytes, as active homeostatic partners of neurons. Beyond saltatory conduction, myelin participates in experience-dependent circuit tuning (adaptive myelination), antioxidant defense, and direct metabolic support of axons (Mount & Monje, 2017; Bechler et al., 2018; Z. Huang et al., 2025). These functions position oligodendrocytes not only as structural allies, but also as sensors and effectors that stabilize neuronal function across fluctuating energetic demands. While the central nervous system (CNS) is often portrayed as relying primarily on glucose and lactate for ATP, recent work indicates that oligodendroglial lipid metabolism is more dynamic than previously appreciated and can contribute to the energetic stability of axons. Especially when glucose availability is limited, lipid-rich myelin is mobilized to support energy metabolism (Asadollahi et al., 2024). Together, these observations motivate the central idea of this review: myelin lipid reserves can operate as a conditional metabolic buffer that is tapped when glycolytic supply falters.

The conditional metabolic buffer refers to a reserve capacity that is not constitutively engaged but can be rapidly recruited when energy demand exceeds the ability of canonical substrates (primarily glucose-derived pathways) to sustain ATP production and ionic homeostasis. In the context of myelinated axons, we propose that myelin lipid pools represent a conditional buffer because their mobilization requires a specific convergence of constraints: reduced effective glucose supply, partially preserved oxygen availability, and sufficient mitochondrial capacity to metabolize lipid-derived substrates without causing disproportionate oxidative damage. Mechanistically, mobilizing the buffer is expected to involve lipid mobilization, intracellular routing of fatty acids through mitochondrial and peroxisomal pathways, and metabolic partitioning that spares glucose for axons while meeting oligodendrocyte energy needs. Importantly, lipid buffering is not assumed to “replace” glucose under all conditions. Rather, it provides contingent support that can prolong axonal viability when glycolytic flux is insufficient (Asadollahi et al., 2024).

This perspective is particularly relevant to disorders characterized by cerebral hypometabolism. In Alzheimer’s disease (AD), reductions in glucose uptake and utilization, deficits in glucose transporter 1 (GLUT1/GLUT3), impaired pyruvate dehydrogenase and α-ketoglutarate dehydrogenase activity, and stress-activated AMP-activated protein kinase (AMPK) signaling converge on an energy-deficient neural milieu that precedes clinical symptoms by years (Connolly et al., 2019; Gibson et al., 1988, 2012; Kumar et al., 2022; Yuan et al., 2024). Acute energy crises such as ischemic stroke pose a different but related challenge. In the ischemic core, ATP is rapidly exhausted. In the penumbra, persistent glucose delivery in the setting of inadequate oxygen can drive lactic acidosis and oxidative stress, complicating simple substrate supplementation strategies (Robbins & Swanson, 2014). Oligodendrocytes are highly vulnerable to hypoxic–hypoglycemic injury due to iron load, low glutathione, and lipid-rich membranes, yet they also accumulate in the penumbra after transient ischemia and may contribute to early metabolic support and later repair (S. Huang et al., 2023; D. Chen et al., 2020; Nishiyama et al., 2009). Determining whether lipid-derived fuels can be leveraged safely in these diseases without worsening acidosis or oxidative injury will be key to converting the buffer concept into practice.

In this review, we synthesize emerging work that recasts myelin as both armor and reservoir to stabilize axonal function. We first summarize the multi-protective functions of myelin to neuron, then detail oligodendroglial lipid anabolism and catabolism, and examine how chronic and acute energy failure conditions may recruit or derail this putative buffer. We close with mechanistic gaps, methodological priorities, and translational avenues for safely mobilizing, matching, and replenishing oligodendroglial lipid reserves. By integrating structural biology with metabolism, we aim to clarify when myelin is a resource to be tapped and to outline strategies to convert this insight into disease-modifying interventions.

## Multiple Protective Functions of Myelin: “Armor” of the Neuron

**Fig. 1:**
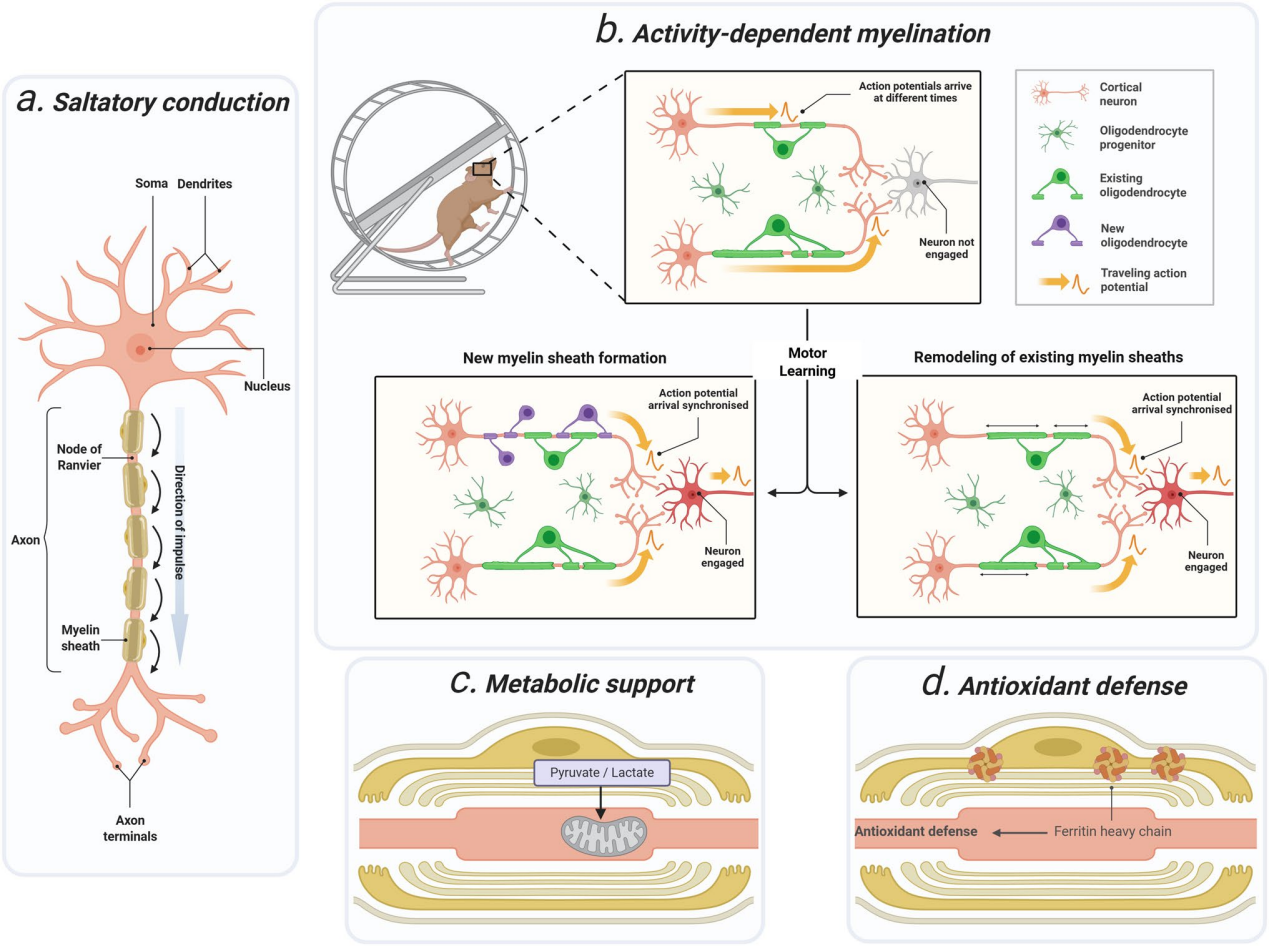
Key protective functions of myelin for neurons. **a** The discontinuous structure of myelin enables saltatory conduction of action potentials, which markedly accelerates signal transmission. **b** During motor learning, sustained neuronal activity drives adaptive myelination in the engaged circuit. This consolidates subsequent responses and increases circuit strength. The change arises from two processes: increased differentiation of oligodendrocytes or increased myelin abundance on existing axons. This function is essential for skill acquisition. Myelination also occurs in activity-independent modes that support baseline circuit maintenance. **c** In addition to astrocytic support, myelin contains transport pathways that deliver energy substrates to axons, providing extra metabolic support. **d** Myelin also confers antioxidant defense, buffering excess iron to protect neurons. Figure created with BioRender

Given the intimate anatomical and functional coupling between axons and myelin (Simons et al., 2014), they engage in multiple essential physiological interactions, including action potential propagation, adaptive myelination, attenuation of neuronal oxidative stress, and metabolic support of neurons (Kedia & Simons, 2025; Z. Huang et al., 2025) (Fig. 1). Beyond its facilitative role in accelerating electrical signal transmission, the latter three homeostatic circuit functions have become the focus of increasingly in-depth investigation in recent years (Kedia & Simons, 2025).

Adaptive myelination refers to the activity evoked adjustment of myelin sheath formation and remodeling, a process that extends into adulthood and underpins both experience-dependent plasticity and normative neurological function (Mount & Monje, 2017). Through this process, the brain undergoes continual experience-dependent remodeling, potentiating or diminishing specific circuits in proportion to practice and the learning of new skills (Mount & Monje, 2017). In response to neuronal activity, adaptive myelination regulates oligodendrocyte lineage dynamics via Neuregulin-ErbB and BDNF pathways, NMDA-receptor and GABAergic signaling, and activity-dependent ATP-LIF transduction, ultimately reshaping myelin sheath biogenesis and architecture (Bechler et al., 2018). Neuroimaging in healthy young adults indicates that, relative to baseline, right-arm visuomotor training significantly elevates myelin content in task-relevant areas, notably the left intraparietal sulcus and parieto-occipital sulcus (Lakhani et al., 2016). While beneficial for healthy cognitive functions (Z. Huang et al., 2025), adaptive myelination can also contribute to maladaptive processes. In mice, morphine enhances adaptive myelination within the ventral tegmental area, a process necessary for opioid reward (Yalcin et al., 2024). With advancing insight into this function, it has emerged as a focal point for mechanistic and translational research in diverse neurological diseases. Importantly, oligodendrocyte generation and myelin formation also occur in an activity-independent manner, contributing to baseline sheath maintenance and neuronal support even when myelination is not explicitly driven by experience-dependent activity (Bonetto et al., 2020). Accordingly, the adaptive (activity-dependent) examples highlighted here are intended to illustrate one prominent mode of axon–glia coupling rather than to imply that myelination is exclusively activity-driven. Because activity-dependent remodeling also elevates metabolic demand and oxidative load, we next discuss how myelin contributes to antioxidant defense and limit redox-driven damage to oligodendrocytes and axons.

While oxidative stress compromises myelin integrity, myelin itself participates in bolstering the nervous system’s antioxidant defense mechanisms (Luoma et al., 2015; Mukherjee et al., 2020). Oxidative stress disrupts cellular metabolism, impedes oligodendrocyte progenitor differentiation, and limits remyelination (Spaas et al., 2021). Interestingly, ferritin heavy chain is enriched in murine oligodendrocytes and released through extracellular vesicle (EV)-dependent unconventional secretion. Loss of myelin-derived EVs or ferritin heavy chain 1 (FTH1) expression yields neuronal loss with oxidative injury, indicating that myelin confers antioxidant protection by sequestering excess iron (Mukherjee et al., 2020). Notably, plasmalogen-deficient myelin is more susceptible to reactive oxygen species (ROS)-driven decompaction (Luoma et al., 2015). During inflammation, reactive oxygen and nitrogen species rise sharply, overwhelming endogenous antioxidant defenses and precipitating oxidative and nitrative stress (Smith et al., 1999).. These protective roles converge on a shared requirement, maintaining energy homeostasis, prompting a closer look at how oligodendrocytes and myelin directly support axonal metabolism through axon–glia coupling.

Oligodendrocytes provide metabolic support to neurons by transferring energy substrates through specialized channels and transporters (Philips & Rothstein, 2017). Oligodendrocytes can metabolically support axons by providing monocarboxylates, classically discussed as lactate, but potentially also pyruvate, to sustain axonal energy demands. Notably, a recent study reported that mature oligodendrocytes display minimal detectable LDH expression and proposed that glycolytic products may be exported predominantly as pyruvate rather than lactate, with LDHA being more evident during development and remyelination (Spate et al., 2024). This metabolic support must be tightly matched to axonal energy demand to avert local acidosis and injury (Looser et al., 2024; Saab et al., 2016). Additional details regarding neuronal metabolic support are discussed in subsequent sections. Accordingly, myelin helps maintain neuronal homeostasis at multiple levels, functioning as an armor-like sheath. This role relies on a rich array of sensors that detect deviations in molecular concentrations (e.g., oxygen, glucose, lipids, and ions). Once a change is detected, diverse effector mechanisms translate it into specific responses aimed at restoring the homeostatic set point (Kedia & Simons, 2025).

## Oligodendroglial Lipid: “Adipose Tissue” in the Brain

Having outlined how myelin protects axons as an “armor”, we next focus on the biochemical basis that enables myelin to function as a metabolic “reservoir”. Because the sheath is exceptionally lipid-rich, understanding its lipid composition and turnover is essential for explaining how a conditional buffer could be engaged under energy stress.

**Fig. 2:**
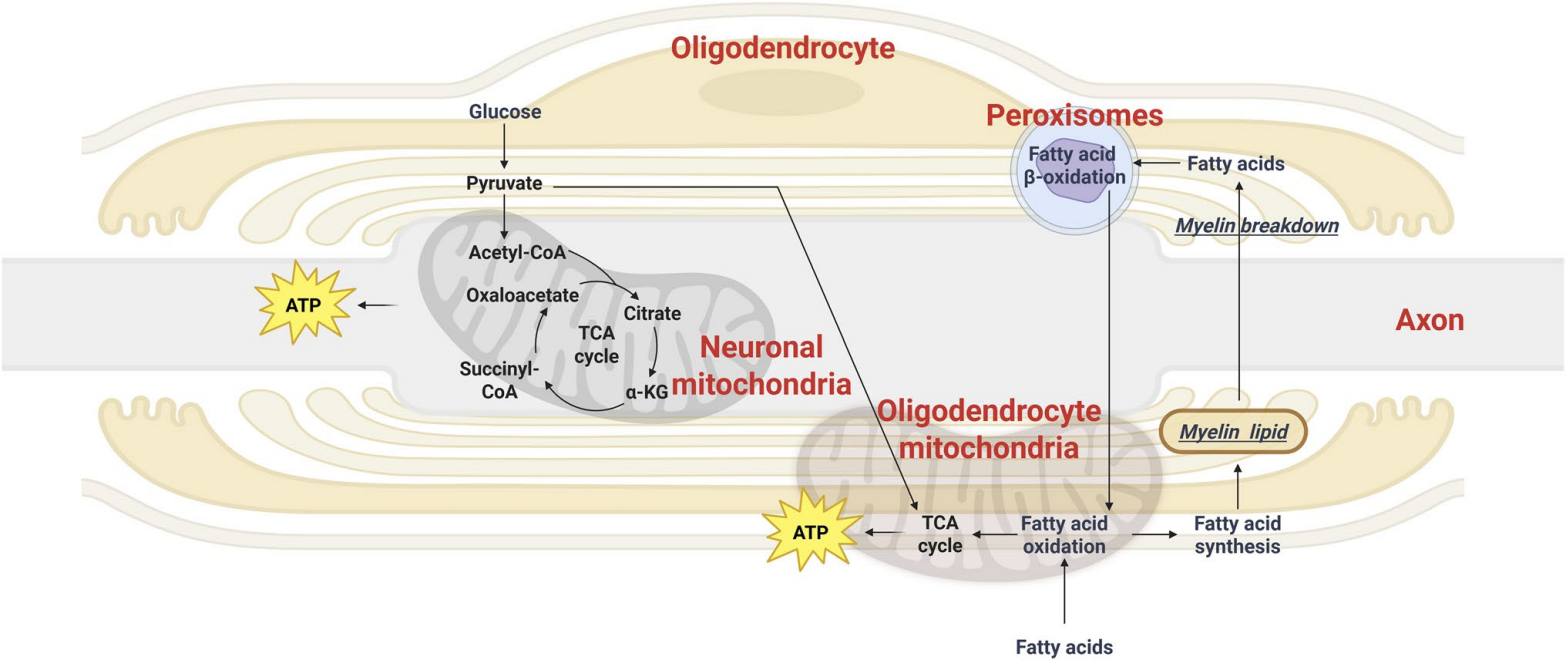
Myelin supports axonal energy metabolism via carbohydrate and lipid oxidation. Neuronal energy is largely supported by glucose-derived lactate/pyruvate supplied through astrocytic metabolism, and myelin lipid turnover provides an additional conditional buffer that can reduce oligodendrocyte glucose dependence and preserve fuel availability for axons. Myelin forms a lipid-based energy buffer. During normal myelin turnover, lysosomal breakdown of myelin lipids releases fatty acids. These fatty acids enter β-oxidation in mitochondria and peroxisomes, supplying precursors for new myelin lipid synthesis. When glucose use declines, myelin lipid synthesis decreases. fatty acids-derived acetyl-CoA is generated to support mitochondrial respiration and maintain oligodendrocyte survival. The shift from routine turnover to lipid-based ATP production spares more glucose-derived pyruvate and lactate for axons. The axonal compartment can then sustain ATP production and limit axonal degeneration. Myelin mitochondria also oxidize imported fatty acids or channel them into upstream steps for myelin lipid synthesis. Figure created with BioRender

Neural energy homeostasis emerges from coordinated substrate routing across neurons/axons, astrocytes, and oligodendrocytes. Neuronal energy is primarily supplied by blood‐derived glucose and its metabolite lactate, whereas astrocytes provide a major buffering node through glycogen mobilization during hypoglycemia and are widely regarded as the predominant site of fatty acid oxidation in the adult brain (Morant-Ferrando et al., 2023). However, recent evidence indicates that myelin lipid metabolism can also furnish ATP to neurons (Asadollahi et al., 2024). The myelin sheath is characterized by an exceptionally high lipid content (70%–85%) and correspondingly low protein composition (15%–30%), whereas most biological membranes exhibit an approximately equal distribution of lipids and proteins (Williams & Deber, 1993). Cholesterol is highly enriched in myelin, representing the most abundant lipid in the CNS and accounting for approximately 46% of myelin lipids (Barnes-Velez et al., 2023). It is located within the phospholipid bilayer, where it stabilizes membrane proteins and helps maintain membrane fluidity and permeability (Lingwood & Simons, 2010; S. T. Yang et al., 2016). It is well established that upregulating the activity of cholesterol biosynthesis–related sterol regulatory element-binding protein 1 (SREBP-1) markedly enhances oligodendrocyte myelinogenesis (X. Liu et al., 2024). For the maintenance of cholesterol, proteolipid protein (PLP) is essential for maintaining cholesterol homeostasis in myelin and also contributes to the adhesion and stabilization of the myelin’s inner and outer leaflets (Werner et al., 2013). Additionally, galactocerebroside (GalC), which constitutes approximately 17% of myelin lipids, promotes myelin maintenance and nodal organization, while plasmalogens, accounting for roughly 13%, are critical for myelin formation and structural integrity. Fatty acids serve as basic building blocks for all major myelin lipid classes, and fatty acid metabolism plays an important role of lipid metabolism in maintaining myelin homeostasis (Barnes-Velez et al., 2023) (Fig. 2).

### Anabolic Lipid Metabolism In Oligodendrocytes

The unique lipid architecture imposes a continuous biosynthetic demand for sheath maintenance; therefore, we summarize the anabolic routes and substrate sources that sustain myelin lipid renewal. Anabolic lipid metabolism in oligodendrocytes is highly dynamic. In adulthood, myelin-associated proteins are not static but instead undergo continuous dynamic turnover, requiring a steady supply of newly synthesized proteins to replace those that are degraded (Meschkat et al., 2022). Similarly, myelin lipids undergo dynamic renewal to better preserve sheath integrity. Notably, in older individuals with the increased lipid turnover rate, suggesting that myelin becomes more susceptible to damage with aging (Ando et al., 2003). Oligodendrocytes typically synthesize most lipids de novo (Poitelon et al., 2020). Glucose is converted via glycolysis to pyruvate, which enters mitochondria and becomes acetyl‐CoA. Intermediates like citrate are exported to the cytosol where fatty acid synthase generate fatty acids and complex lipids (Nave et al., 2017). However, in mice, oligodendrocytes lacking the pyruvate dehydrogenase still form and maintain normal myelin sheaths (Della-Flora Nunes et al., 2017). This indicates that pyruvate‐derived acetyl‐CoA is not essential for myelin lipid synthesis or maintenance. Interestingly, some downstream metabolites of the lactate shuttle in axons return to oligodendrocytes to fuel lipid synthesis and contribute to myelin formation (Nave et al., 2017; L. Liu et al., 2017). Although most cholesterol in oligodendrocytes is derived from de novo synthesis, under conditions of increased demand it can be supplemented by uptake of extracellular sterols via low-density lipoprotein receptor-related protein 1 (LRP1) (Lin et al., 2017; Saher & Simons, 2010; Saher et al., 2011). Notably, choline transporter-like proteins (CTLs), especially CTL1 (SLC44A1) and CTL5 (SLC44A5), mediate high-affinity choline uptake in oligodendrocytes, a process essential for the biosynthesis of choline-derived plasmalogens (Traiffort et al., 2013). This pathway is critical for myelin formation, as deletion of these transporters in oligodendrocytes severely impairs myelin sheath development (M. C. Liu et al., 2025). In addition to circulating ingredients, astrocytes also act as a relay station for fatty acid transfer. Astrocyte‐specific knockout of the SREBP cleavage‐activating protein (SCAP) gene in mice dramatically reduces brain cholesterol levels, which further demonstrate that astrocyte‐derived lipid is helpful for myelin formation and maintenance (Camargo et al., 2017). Remarkably, hyperactive neurons release excess fatty acids via ApoE‐containing lipoproteins, which astrocytes then detoxify by upregulating lipid‐peroxidation enzymes. This coupling prevents neuronal fatty acid accumulation and protects against lipotoxicity (Ioannou et al., 2019). Therefore, oligodendrocytes may likewise mitigate neuronal lipotoxicity by absorbing fatty acids and engaging in lipidic anabolism, although more direct evidence is required. Together, these anabolic pathways support routine renewal and growth of myelin; we next turn to the catabolic arm, lipid turnover and β-oxidation, to explain how the same lipid pools can be mobilized as a conditional buffer during energy deprivation.

### Lipid Catabolism in Oligodendrocytes

Prevailing theories suggest that the central nervous system exhibits a limited reliance on fatty acid β-oxidation as a primary energy-generating pathway due to several intrinsic constraints. These include the markedly elevated oxygen demand associated with β-oxidation, the excessive generation of ROS that can exacerbate oxidative stress, and the comparatively slow kinetics of ATP production relative to glucose metabolism (Schonfeld & Reiser, 2013; Z. Chen et al., 2024). Furthermore, oligodendrocytes, as the principal myelinating cells of the central nervous system, prioritize the synthesis of fatty acids for the continual production and maintenance of myelin lipids rather than their catabolic degradation for energy provision (Barnes-Velez et al., 2023; Schonfeld & Reiser, 2013). Approximately 20% of the total energy expenditure of the adult brain is attributed to fatty acid oxidation. It is generally accepted that this oxidative process occurs predominantly, if not exclusively, within astrocytes (Panov et al., 2014). Moreover, astrocytes play a pivotal role in sustaining neuronal energy homeostasis through their glycogen reserves. During episodes of hypoglycemia, these glycogen stores are mobilized and converted into metabolic substrates that are subsequently transferred to neighboring neurons or axons, where they are aerobically metabolized to support energy production (Brown & Ransom, 2007). This highlights the critical function of astrocytes as metabolic supporters and energy reservoirs for the central nervous system. However, the energetic profile of myelinating cells could resemble that observed in astrocytes, and they likewise participate in axon–glia metabolic coupling (Poitelon et al., 2020; Verheijen et al., 2003). Notably, in the context of lipid metabolism, continuous oligodendroglial lipid turnover provides a critical energy reserve for sustaining the function and integrity of white matter tracts (Asadollahi et al., 2024; Funfschilling et al., 2012). The uptake fatty acids are transported into mitochondria, where they undergo β-oxidation and enter the tricarboxylic acid cycle to generate ATP. Simultaneously, the acetyl-CoA produced during this process can serve as a precursor for de novo fatty acid synthesis within the cell (Poitelon et al., 2020).

Myelinating cells are capable not only of taking up circulating fatty acids for catabolic metabolism but also of degrading their own myelin-derived lipids under conditions of severe energy deprivation. A recent study revealed that oligodendrocytes exhibit greater survival than astrocytes during prolonged glucose deprivation in mice, suggesting the presence of an intrinsic energy reserve mechanism. This metabolic resilience is primarily attributed to fatty acid β-oxidation within both mitochondria and peroxisomes. Under hypoglycemic conditions, myelin-derived lipids undergo catabolic breakdown to release FAs, which are subsequently oxidized to generate acetyl-CoA and ATP. Then, this lipid-driven energy supply is sufficient to maintain basal oligodendrocytes viability and preserve axonal conduction. Pharmacological inhibition of mitochondrial β-oxidation (e.g., with 4-bromocrotonic acid) or peroxisomal β-oxidation (e.g., with thioridazine) under glucose-deprived conditions markedly compromises oligodendrocytes survival and optic nerve conduction, underscoring the central role of this catabolic pathway in white matter energy homeostasis. In addition, electron microscopy of glucose-deprived optic nerves revealed an increased g-ratio (defined as the inner axonal radius divided by the radius of the axon plus myelin) and evidence of autophagy-mediated myelin degradation. These observations indicate that reduced glucose availability shifts the balance of myelin turnover toward degradation. Furthermore, basal autophagy in oligodendrocytes is upregulated during glucose deprivation, facilitating lipid mobilization for β-oxidation, although the master autophagy regulator TFEB appears to be largely dispensable for this process. Further experiments involving conditional ablation of the glucose transporter GLUT1 resulted in progressive myelin loss in vivo, without overt axonal pathology or behavioral deficits. This phenotype reflects the consequence of sustained energy deprivation, highlighting that continuous lipid catabolism within oligodendrocytes can support short-term survival at the expense of long-term myelin integrity (Asadollahi et al., 2024). This phenomenon may explain why metabolic dysfunction often precedes the onset of structural lesions in disorders such as AD and vascular dementia (Klemmensen et al., 2024). Oligodendrocytes may, akin to astrocytes, exploit fatty acid–derived acetyl-CoA for ketone body production within the cholesterol biosynthetic pathway, either through the action of 3-hydroxy-3-methylglutaryl-CoA lyase or via the direct deacylation of acetoacetyl-CoA (Auestad et al., 1991). A ketogenic diet significantly enhances fatty acid metabolism and ketone body levels in rats with neural injury, markedly reducing the extent of myelin loss (Mu et al., 2022). Further in-depth experimental investigations are required to substantiate this possibility. A central signaling node that can couple energy deficit to lipid mobilization and β-oxidation is AMPK (Herms et al., 2015). Under glucose deprivation or increased ATP demand, AMPK activation is expected to shift oligodendrocytes from an anabolic, myelin-maintenance program toward a catabolic state that supports survival. Mechanistically, AMPK can promote fatty acid utilization by phosphorylating acetyl-CoA carboxylase (ACC), lowering malonyl-CoA levels and thereby relieving inhibition of mitochondrial fatty-acid import (CPT1-dependent entry), which facilitates β-oxidation. In parallel, AMPK can promote autophagy initiation (e.g., via ULK1 activation and mTORC1 restraint), providing a route for lipophagy and mobilization of lipid substrates that feed mitochondrial and peroxisomal oxidation (He et al., 2020). Within our “conditional buffer” framework, AMPK therefore represents a plausible molecular trigger for the transition from baseline myelin turnover to buffer engagement during energetic stress. Overall, these findings provide mechanistic insight into the white matter abnormalities observed in conditions characterized by cerebral hypometabolism.

More broadly, oligodendrocytes exhibit metabolic flexibility and can re-route substrate use under energy stress, consistent with a regulated switch between anabolic myelin maintenance and catabolic support (Asadollahi et al., 2024). However, key uncertainties remain, including the quantitative in vivo contribution of different substrates, which lipid pools are preferentially mobilized, and how fuel transfer is routed within the axon–myelin unit during disease.

The concept that lipid catabolism in oligodendrocytes functions as a metabolic buffer appears to represent an evolutionarily conserved capability. In nonmyelinating species, such as lampreys and Drosophila, axon-associated glial cells contain abundant lipid droplet reservoirs that are mobilized under conditions of energy scarcity. This observation suggests that radial axonal sorting, ensheathment, and potentially metabolic support are ancient glial functions that predate the evolutionary emergence of myelin in jawed vertebrates (Silva et al., 2022; Weil et al., 2018). Interestingly, during hibernation, the myelin of the Syrian hamster brain appears to achieve cold-adaptive membrane stability and functionality primarily through selective remodeling of its lipid composition—particularly in the species of fatty acids and structural modifications of plasmenylethanolamines (Blaker & Moscatelli, 1978). Similarly, in the sciatic and peroneal nerves of starved rats, Schwann cells— which share functional roles with oligodendrocytes in myelin lipid metabolism—exhibit morphological instability or structural remodeling of the myelin sheath, accompanied by focal demyelinating changes (Boucanova & Chrast, 2020; Collins et al., 1964). Human studies suggest that under conditions of extreme metabolic stress, such as glycogen depletion, myelin lipids may be mobilized as a local energy reserve, resulting in a transient reduction of myelin content—a phenomenon referred to as metabolic myelin plasticity. Evidence from marathon runners indicates that the myelin water fraction in specific white matter tracts markedly decreases within 1–2 days post-race, followed by partial recovery at two weeks (without full normalization), and a complete return to baseline across all affected regions by two months. These observations indicate that the alterations are transient and reversible (Ramos-Cabrer et al., 2025). In contrast, long-term exercise training, rather than acute single-session activity, significantly enhances the expression of myelin-associated protein myelin basic protein (MBP), regardless of whether the regimen involves aerobic exercise or high-intensity interval training (Feng et al., 2023; C. Wu et al., 2025). In summary, evidence across species suggests that under hypoglycemic or low-glucose conditions, myelin turnover can function analogously to adipose tissue mobilization, temporarily supplying energy to sustain axonal metabolism. This metabolic feature may help explain the progressive myelin loss observed in neurodegenerative disorders characterized by underlying metabolic insufficiency.

While myelin lipid mobilization may serve as a conditional metabolic buffer during energetic stress, it also entails intrinsic risks because the buffer substrate is a structural component of the axon–glia unit. Experimental glucose deprivation increases g-ratio and shows autophagy-mediated myelin degradation, indicating that reduced glucose availability can shift myelin turnover toward net breakdown (Asadollahi et al., 2024). Accordingly, any strategy that amplifies lipid mobilization or β-oxidation risks myelin thinning and demyelination, potentially impairing conduction and increasing axonal vulnerability over time. This trade-off is consistent with in human evidence that sustained energetic stress can lead to progressive myelin loss even in the absence of overt axonal pathology (Ramos-Cabrer et al., 2025), suggesting that metabolic compensation may preserve short-term function at the expense of long-term myelin integrity. In addition to structural loss, enhanced fatty acid oxidation can impose redox and inflammatory liabilities. Mitochondrial and peroxisomal β-oxidation can elevate reactive oxygen species, and myelin breakdown may generate bioactive lipid intermediates that can amplify neuroinflammation (Tiwari & Simons, 2025). Therefore, the therapeutic rationale is not to indiscriminately “accelerate myelin breakdown,” but to define the boundary conditions under which buffer engagement is expected to be beneficial.

## Energy Metabolism Dysregulation in CNS: Chronic and Acute

### Alzheimer’s Disease

AD is a latent, progressive, and irreversible neurodegenerative disorder characterized by the accumulation of amyloid-β (Aβ) and the hyperphosphorylation of tau protein (Zou et al., 2023; C. Wu et al., 2023). Impaired cerebral energy homeostasis has been strongly implicated in the pathogenesis of AD, as reflected by diminished glucose uptake and metabolism, disruptions in insulin signaling cascades, and deficits in mitochondrial function (Yuan et al., 2024). Notably, in individuals with late-onset AD, intrinsic abnormalities in energy metabolism are evident across multiple levels within neural progenitor cells and astrocytes. These defects, compounded by impaired signaling and substrate availability as well as dysregulated transcriptional control, collectively establish a vulnerable metabolic foundation predisposing the brain to neurodegeneration (Ryu et al., 2021). Considering that late-onset cases account for the vast majority (90–95%) of AD, this dimension represents a critical area for further exploration (Harman, 2006). Encouragingly, this energy dysfunction appears to precede clinical symptoms by many years, underscoring its potential as an early biomarker for disease prediction and intervention (Connolly et al., 2019).

Metabolomic analyses have confirmed that dysregulated energy metabolism constitutes a core feature of AD pathology. Compared to controls, the AD brain exhibits a systematic reduction in glucose-derived metabolites, including those involved in glycolysis and the pentose phosphate pathway, as well as in the ketone body β-hydroxybutyrate. These findings highlight the potential value of targeting glucose and ketone utilization pathways for both metabolic interventions and biomarker development (Patel et al., 2024). The reduction in cerebral glucose utilization observed in AD is closely associated with diminished levels and expression of critical glucose transporters, namely GLUT1 and GLUT3 (Kumar et al., 2022). Beyond the reduced availability of energy substrates, enzyme deficiencies also play a significant role in the impaired energy metabolism characteristic of AD (Gibson et al., 2012). The pyruvate dehydrogenase complex (PDHC), which serves as the critical enzymatic bridge between glycolysis and the tricarboxylic acid cycle, has been found to be markedly deficient in postmortem brain specimens from Alzheimer’s disease patients (Gibson et al., 1988). As a central enzyme in the tricarboxylic acid cycle, α-ketoglutarate dehydrogenase complex (KGDHC) dysfunction may alter mitochondrial metabolite profiles or trigger the release of specific mitochondrial signaling molecules. These signals, in turn, can modulate calcium channels on the endoplasmic reticulum (ER), and prolonged dysregulation of this feedback loop increasingly contributes to calcium signaling abnormalities resembling those observed in the AD phenotype (Gibson et al., 2012). Specifically, prolonged inhibition of KGDHC in both neuroblastoma cells and primary neurons has been shown to enhance the calcium response to potassium-induced depolarization by approximately 28–43% following 24 to 48 h of treatment (Gibson et al., 2012). Regarding enzymatic activity, the heightened oxidative stress characteristic of Alzheimer’s disease can inactivate key glycolytic enzymes (Butterfield & Lange, 2009). More critically, elevated AMPK phosphorylation has been documented in both Alzheimer’s disease patients and corresponding mouse models, reflecting its activation under energy-deficient conditions, moreover, AMPK can directly phosphorylate tau at multiple sites, thereby exacerbating tau pathology and advancing disease progression (Yu et al., 2022). Beyond tau-directed effects, sustained AMPK activation in an energy-deficient milieu is also positioned to remodel cellular fuel choice, favoring lipid mobilization and oxidative substrate routing. In the context of our buffer hypothesis, this provides a mechanistic link by which chronic hypometabolism could recruit oligodendroglial lipid reserves to stabilize function. However, prolonged AMPK signaling may also suppress lipid and cholesterol synthesis programs needed for myelin maintenance and remyelination, potentially contributing to a stage-dependent transition from compensation to myelin vulnerability (Y. Li et al., 2024; Owaki et al., 2023). In summary, Alzheimer’s disease–associated neurodegeneration is characterized by substrate depletion and diminished enzymatic efficiency, reflecting a broader dysregulation of energy metabolism. However, the causal relationships among these metabolic derangements and their viability as therapeutic targets remain elucidated.

Abnormal lipid accumulation was noted in early neuropathological studies of AD, and in recent decades, lipid dyshomeostasis has emerged as a central focus in the investigation of AD pathogenesis (Yin, 2023). However, whether lipid droplet accumulation is protective or deleterious remains a matter of ongoing debate. Glial lipid droplets can serve a cytoprotective function by sequestering hydrophobic and potentially lipotoxic species, with biogenesis in the aging brain driven by triggers such as chronic inflammation and oxidative insult (Farmer et al., 2020; Unger & Orci, 2002). Conversely, excessive lipid droplet accumulation within astrocytes and microglia is frequently associated with activation of pro-inflammatory transcriptional programs and release of neurotoxic mediators, thereby contributing to neuronal dysfunction and injury (Ralhan et al., 2021). Despite differences among individual species, the total fatty acid level in the cerebrospinal fluid of AD patients is elevated relative to that of healthy controls (Fonteh et al., 2014). Also, elevated cholesterol levels augment the activity of both β- and γ-secretases, enzymes essential for Aβ generation, thereby further exacerbating AD pathology (Marzolo & Bu, 2009). Furthermore, ApoE4 is a major genetic driver of lipid dyshomeostasis in late-onset AD and can affect myelin stability through convergent, cell-type-specific mechanisms. Ex vivo assessments of hippocampal fatty acid β-oxidation in acute brain slices from young ApoE4 knock-in mice reveal impaired lipid catabolism coupled with marked lipid accumulation, indicative of a sustained deficit in β-oxidative metabolism (Qi et al., 2021b). Astrocytes, the canonical mediators of cerebral lipid catabolism, exhibit markedly impaired lipid metabolism in AD with ApoE4. It leads to excessive accumulation of fatty acids and cholesterol within astrocytes and their associated neurons, culminating in pervasive lipid dyshomeostasis (L. G. Yang et al., 2023). Under ApoE4 expression, neurons compensate for the loss of astrocytic metabolic support by upregulating their own fatty acid β-oxidation. However, this adaptive response further imposes mitochondrial stress and elevates reactive oxygen species production (Qi et al., 2021a). Accordingly, strategies to enhance the metabolism or efflux of lipids within neurons and glia have emerged as a promising therapeutic avenue in AD (Blanchard et al., 2022; Mota et al., 2021; Nordestgaard et al., 2015).

As discussed above, oligodendrocytes appear capable of upregulating lipid biosynthesis to support myelin formation, thereby functioning as intracellular lipid relay stations. Nonetheless, it remains unclear whether oligodendrocytes can internalize lipid accumulations originating from neurons, even though astrocyte-derived lipids have been demonstrated to be essential for myelin formation (Camargo et al., 2017). Available evidence supports the view that oligodendrocytes in ApoE carriers exhibit intrinsically elevated cholesterol levels, however, whether this reflects a pathological process or a compensatory adaptation remains unresolved (Blanchard et al., 2022). Pharmacological enhancement of oligodendrocyte lipid efflux has been shown to rescue myelination deficits in Alzheimer’s disease models (Blanchard et al., 2022). Together, these findings support a model in which ApoE4-driven lipid dysregulation yields a brittle myelin state, structurally and metabolically less robust, thereby increasing vulnerability to oxidative stress and reducing the tolerance for prolonged lipid-buffer mobilization during chronic hypometabolism. Theoretically, accelerating myelin turnover could both mobilize accumulated lipids for metabolic use and supply essential energy substrates, making this an underexplored yet potentially promising therapeutic target in AD.

In summary, AD is characterized by a long preclinical window of progressive cerebral hypometabolism in which glucose-derived energy becomes increasingly insufficient relative to network demands. Under our framework, this is a canonical setting predicted to mobilize the conditional lipid buffer: oligodendrocytes may increase lipid mobilization and oxidative routing to preserve their own energetic stability and to spare glucose for axonal function. The conditional metabolic buffer hypothesis may predict two complementary trajectories across disease stage. Early, buffer recruitment may be adaptive and partially compensatory. Later, buffer failure may emerge from worsening myelin structural instability, impaired lipid processing, and redox overload, contributing to lipid droplet accumulation and white matter vulnerability. This disease–buffer linkage generates testable predictions. Early AD should show oligodendrocyte signatures consistent with energetic stress sensing and lipid mobilization and progression should correlate with impaired lipid handling and oxidative injury. Notably, enhancing lipid utilization or myelin turnover in AD should be approached with caution, as chronic activation of lipid-buffer pathways may gradually compromise myelin integrity and promote demyelination, necessitating stage-specific strategies that balance short-term metabolic support with long-term sheath maintenance.

### Ischemia Stroke

Ischemic stroke, which accounts for approximately 87% of all stroke cases, arises from reduced cerebral blood flow caused by arterial occlusion, cardiac arrest, vasospasm, or other etiologies, ultimately resulting in insufficient delivery of oxygen and nutrients to brain tissue (Hilkens et al., 2024; Lui et al., 2025). It represents the most common cause of long-term disability, the second leading cause of dementia, and the fourth leading cause of mortality in developed countries (Sveinsson et al., 2014). The pathological features of ischemia stroke are characterized primarily by neuronal apoptosis, neuroinflammatory responses, disruption of the blood–brain barrier (BBB), and glutamate-mediated excitotoxicity (Howard et al., 2024; Wu et al., 2024). Most notably, these pathological processes are accompanied by mitochondrial dysfunction, which reduces ATP production and perturbs the intracellular milieu, ultimately culminating in necrotic cell death (Lei et al., 2025). Following stroke, neurons in the ischemic core undergo irreversible death within minutes, whereas those in the surrounding penumbra progressively degenerate over the subsequent hours to days (Fifield & Vanderluit, 2020). This underscores the critical importance of restoring energy supply during the early stages following stroke. However, glucose supplementation alone does not appear to represent an optimal strategy (Robbins & Swanson, 2014). In the ischemic core, complete or near-complete cessation of blood flow leads to depletion of both glucose and oxygen, accompanied by ATP exhaustion and mild acidosis. Additional hyperglycemia slightly exacerbates acidosis within the core, since only the glucose present at the onset of ischemia can be metabolized. By contrast, in the penumbral region, where residual collateral blood flow persists, there is continuous delivery of glucose, owing to its molar excess in arterial blood, whereas oxygen delivery remains insufficient. This sustained glucose supply drives glycolysis, which may attenuate ATP depletion but simultaneously induces lactic acidosis in proportion to blood glucose levels. During reperfusion, pH levels return to normal and ATP is restored in regions of viable tissue, however, the enhanced glucose influx also augments the generation of ROS (Robbins & Swanson, 2014). This underscores the importance of not only supplementing energy but also effectively regulating the accumulation of metabolic by-products.

Pharmacological modulation of fatty acid metabolism has been proposed to influence cellular energy homeostasis in the bilateral carotid artery occlusion model of stroke, thereby potentially conferring neuroprotection (McCullough et al., 2005). Interestingly, under 24-h glucose deprivation, lipid-rich oligodendrocytes sustain neuronal survival and basic function by mobilizing their own fatty acids to generate ATP. In contrast, astrocytes as the primary energy providers, undergo extensive cell death under glucose deprivation. Although lipid oxidation is accompanied by substantial ROS generation, the death of glucose-deprived optic nerve glial cells is not attributable to oxidative stress (Asadollahi et al., 2024). Lipid mobilization may be most likely to worsen ROS during reperfusion, when restored oxygen delivery and enhanced glucose influx augment ROS generation in viable tissue. Available data suggest that under hypoxic–hypoglycemic conditions of ischemic stroke, oligodendrocytes are particularly vulnerable owing to their high iron load, low levels of reduced glutathione, elevated oxidative metabolic rate, abundant lipid and sphingolipid content, and the high permeability of their glutamate receptors (S. Huang et al., 2023; D. Chen et al., 2020). The resulting myelin disruption or loss can be catastrophic, leading to axonal instability, compromising neuronal viability, and promoting long-term neurological dysfunction (Y. J. Cheng et al., 2024). Given their critical role in energy provision, oligodendrocytes and their progenitor cells are found to increase in the penumbral region following transient middle cerebral artery occlusion, whereas their numbers markedly decline within the ischemic core after reperfusion (Nishiyama et al., 2009; Tanaka et al., 2001). In some cases, cerebral ischemia is followed by the development of oligodendrogliomas, which may represent a defensive mechanism of self-repair in response to injury (Villa Gonzalez & Perez-Alvarez, 2021). In mouse models, immediate administration of oligodendrocyte precursor cells (OPCs) following cerebral ischemia effectively alleviates edema and reduces infarct volume, while promoting neurorestoration after ischemic stroke through activation of the Wnt/β-catenin pathway (Wang et al., 2020). With respect to prognosis, OPC transplantation at 7 days post-ischemic injury has been shown to attenuate brain atrophy and enhance functional recovery in ischemic stroke mice (W. Li et al., 2021). In summary, oligodendroglial lipid metabolism may serve as a potential transient energy source during acute energy deprivation. However, in the context of stroke, where oxygen supply is limited, whether it can exert a similar compensatory effect and how to maximize its contribution remain to be further elucidated. In the anoxic ischemic core, lipid oxidation cannot substitute for ATP production because oxidative metabolism is fundamentally limited. However, in the ischemic penumbra, where oxygen delivery is reduced but not absent, and during early reperfusion, the buffer may be mobilizable and potentially protective. Here, lipid mobilization could support oligodendrocyte survival and maintain axonal integrity by providing alternative energetic routes or sparing glucose during a period of unstable substrate supply. At the same time, reperfusion introduces a high-risk redox environment, and excessive lipid oxidation may amplify ROS (C. L. Chen et al., 2022). Thus, the conditional metabolic buffer hypothesis predicts a narrow therapeutic window in which enhancing controlled lipid utilization or improving lipid handling capacity could be beneficial, whereas indiscriminate stimulation of fatty acid oxidation may be harmful if antioxidant capacity or mitochondrial function is insufficient. This framing explicitly links stroke pathophysiology to the buffer concept and clarifies why benefits may be greatest in penumbral and reperfusion contexts rather than the infarct core. Notably, interventions aimed at mobilizing lipid reserves must explicitly account for demyelination risk and redox load, with potential benefit restricted to contexts where oxidative metabolism is still feasible (e.g., penumbra/early reperfusion), rather than the anoxic core.

## Future Perspectives

The central hypothesis emerging from current evidence is that oligodendroglial lipid reserves operate as a conditional metabolic buffer: they can be mobilized to sustain axonal ATP production when glycolytic supply falters, provided that redox burden, iron handling, and local pH remain within safe bounds. Several mechanistic gaps now define the research agenda. First, the field needs quantitative criteria for “buffer engagement”: when oligodendrocytes switch from anabolic renewal to catabolic fuel release, which lipid pools are tapped (membrane phospholipids vs. stored neutral lipids), and how mitochondrial versus peroxisomal β-oxidation is apportioned. A critical next step is to define oligodendrocyte-wide metabolic state transitions in vivo, particularly how oxygen availability, redox buffering, and iron handling constrain the balance between glycolysis-forward programs and lipid-driven catabolic modes across disease stages. Second, substrate routing from glia to axon must be resolved in vivo with time–space precision, identifying the carriers (e.g., lactate, ketone bodies), the conduits (specialized channels/transporters), and the feedback rules that match flux to axonal demand to avoid focal acidosis. Finally, activity dependent myelination needs to be parsed into adaptive windows that support learning and recovery versus maladaptive reinforcement of pathological circuits, with molecular checkpoints that can be therapeutically engaged.

Progress will hinge on methods that read out metabolism and structure together. Longitudinal white matter resolved fluxomics, combining 13C tracers with high-field magnetic resonance spectroscopy (MRS) and spatial metabolomics, should map lipid to ATP routing across axon–glia units in vivo (Danzi et al., 2023; Radenkovic et al., 2020). Pairing myelin specific MRI metrics (e.g., myelin water fraction) with proteo-lipidomics may reveal a reversible metabolic myelin plasticity state (Bae et al., 2025). Inducible, lineage restricted perturbations of β-oxidation, autophagy, and lipid transport in oligodendrocytes can separate anabolic renewal from catabolic support without astroglial confounds (Asadollahi et al., 2024). Additionally, closed-loop paradigms that couple neural activity manipulations with simultaneous pH, NADH/FAD, and ATP sensors will define operational safety margins for energy exchange (Z. Wu et al., 2022).

Translationally, in AD and chronic hypometabolic states, strategies should prioritize improving utilization from glial neuronal fuel routing and β-oxidation tuning over simply increasing substrate supply, while restoring sheath stability via plasmalogen support and iron redox control. ApoE4 linked sterol and phospholipid handling in oligodendrocytes warrants targeted correction to prevent a cycle of brittle myelin and inefficient energy transfer. In ischemic stroke, phase-specific regimens are essential. Early reperfusion might pair oxygen delivery with controlled lipid-derived fuels to limit glycolysis driven acidosis, followed by protection of the buffer and, in the subacute/chronic phases, promotion of remyelination and circuit resynchronization. Cell-based augmentation with OPCs, aligned to pro-myelinating cues, could rebuild the anabolic arm once perfusion stabilizes.

## Conclusion

Myelin is more than a part of insulation. As an armor-like interface and an energy buffer, it detects metabolic deviation, allocates fuels, and constrains oxidative and iron stress to stabilize axonal function. The emerging view is not of passive white matter but of a regulated reservoir that can be tapped productively or at a cost when energy fails. Clarifying how to mobilize this reservoir safely, match it to axonal demand, and restore anabolic balance will determine whether oligodendroglial lipid metabolism becomes a tractable lever for disease modification in hypometabolic brain disorders.

## Data Availability

No datasets were generated or analysed during the current study.

## Abbreviations

AD: Alzheimer’s disease;
AMPK: AMP-activated protein kinase;
Aβ: Amyloid-β;
BBB: Blood–brain barrier;
CNS: Central nervous system
CTL1: Choline transporter-like protein 1;
CTL5: Choline transporter-like protein 5;
CTLs: Choline transporter-like proteins;
ER: Endoplasmic reticulum;
EV: Extracellular vesicle;
FTH1: Ferritin heavy chain 1;
GLUT1: Glucose transporter 1;
GLUT3: Glucose transporter 3;
GalC: Galactocerebroside;
KGDHC: α-Ketoglutarate dehydrogenase complex;
LRP1: Low-density lipoprotein receptor-related protein 1;
MBP: Myelin basic protein;
MRS: Magnetic resonance spectroscopy;
OPCs: Oligodendrocyte precursor cells;
PDHC: Pyruvate dehydrogenase complex;
PLP: Proteolipid protein;
ROS: Reactive oxygen species;
SCAP: SREBP cleavage-activating protein;
SREBP: Sterol regulatory element-binding protein
